# Rare renal metastasis: Oligometastatic non-small cell lung cancer diagnosed with ultrasound guided renal biopsy

**DOI:** 10.1016/j.radcr.2025.12.012

**Published:** 2026-01-02

**Authors:** Thomas Neerhut, Thomas McMaster, David Homewood, Gerard Bray, Roger Wilson, Katie Buzacott, Abigail Attwell-Heap

**Affiliations:** a Department of Urology, Sunshine Coast University Hospital, Affiliate Lecturer Deakin University School of Medicine, 6 Doherty Street, Birtinya QLD 4575, Australia; b Department of Urology, Sunshine Coast University Hospital, 6 Doherty Street, Birtinya QLD 4575, Australia; c Department of Radiology, Sunshine Coast University Hospital, RANZCR, Fellow of The European Board of Interventional Radiology (EBIR), 6 Doherty Street, Birtinya QLD 4575, Australia; d Pathology Queensland, The Sunshine Coast University Hospital, 6 Doherty Street, Birtinya Qld, 4575, Australia

**Keywords:** Renal biopsy, Renal metastasis, MDT, NSCLC, PET, Renal mass

## Abstract

Metastatic renal masses present an uncommon diagnostic dilemma. Differentiating malignant metastatic renal lesions from primary renal malignancy relies on a high index of suspicion, multidisciplinary discussion, comprehensive radiological work up and finally, accurate immunohistochemical pathology findings. Here we present a rare case of solitary non-small cell lung cancer metastases to the kidney definitively diagnosed with image guided renal biopsy. We emphasize both the salient radiological features of such masses, as well as the vital role of image guided renal biopsies in such settings. The pivotal role of the radiologist within the oncological multidisciplinary team meeting is also illustrated.

## Introduction

Lung cancer most commonly metastasizes to the brain, bone, liver and adrenal gland. Rarely it may metastasize to the kidney [1]. Only case reports and a limited number of case series describe similar cases, with no formal consensus regarding management [1]. In these rare situations, appropriate recognition and timely diagnosis is pivotal. However, given the rarity of lung cancer metastases to the kidney a high index of suspicion and judicious multidisciplinary (MDT) discussion must be utilized in order to achieve a prompt and accurate diagnosis. Amongst patients with a history of resected non-small cell lung cancer (NSCLC), renal masses are most often identified incidentally following imaging and the rare possibility of metastatic recurrence in the kidney must be considered [2]. An understanding of the typical radiological features of metastatic renal masses when compared to primary renal parenchymal tumors is important. Ultimately, image guided renal biopsy of such masses play a crucial role in achieving timely diagnosis and appropriate management. Here we present a rare case of recurrent oligometastatic NSCLC diagnosed via ultrasound (US) guided renal biopsy. The important role of image interpretation and image guided biopsies in the work up of indeterminate renal masses is emphasized as well as the role of MDT discussion and immunohistochemical techniques.

## Case report

**Fig. 1:**
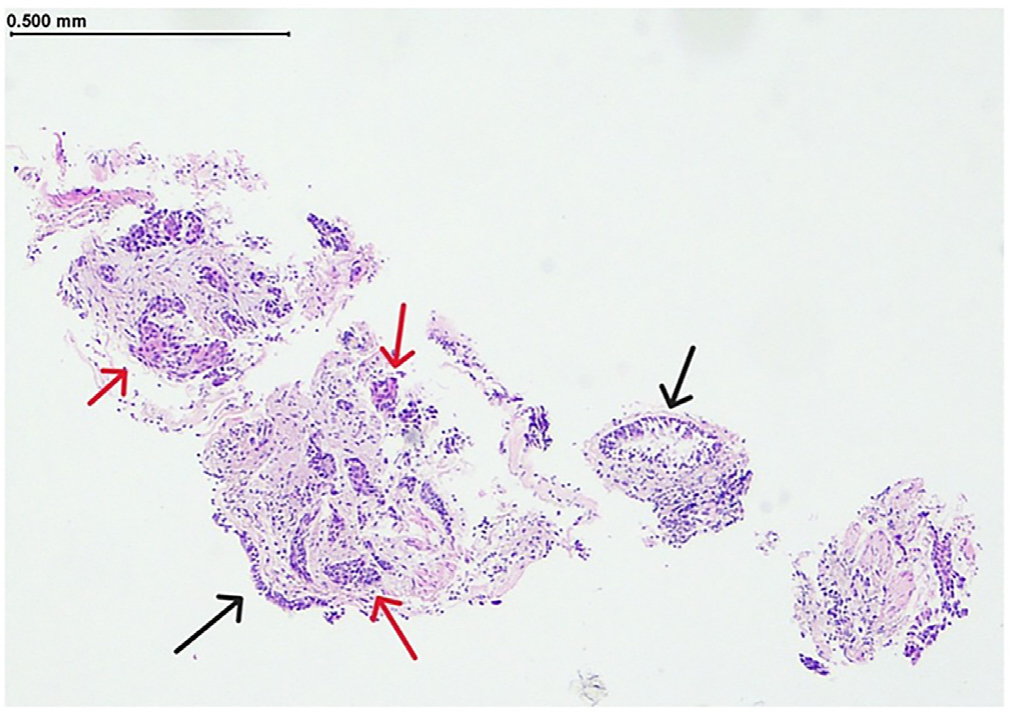
Lung biopsy: Hematoxylin and Eosin staining histopathology showing benign respiratory epithelium (black arrows) with underlying infiltration by sheets of malignant cells (red arrows).

**Fig. 2:**
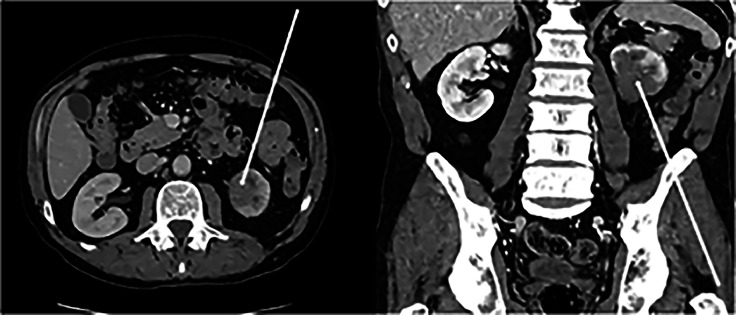
Panel 2a (axial CT image in portal venous contrast phase) demonstrating an endophytic hilar hypodense heterogenous mass measuring 33mm (as indicated by white arrow). Panel 2b (coronal CT in portal venous contrast phase) demonstrating the proximity of the mass extending from the lower pole into to the renal hilum with obliteration of typical hilar anatomy (as indicated by white arrow).

**Fig. 3:**
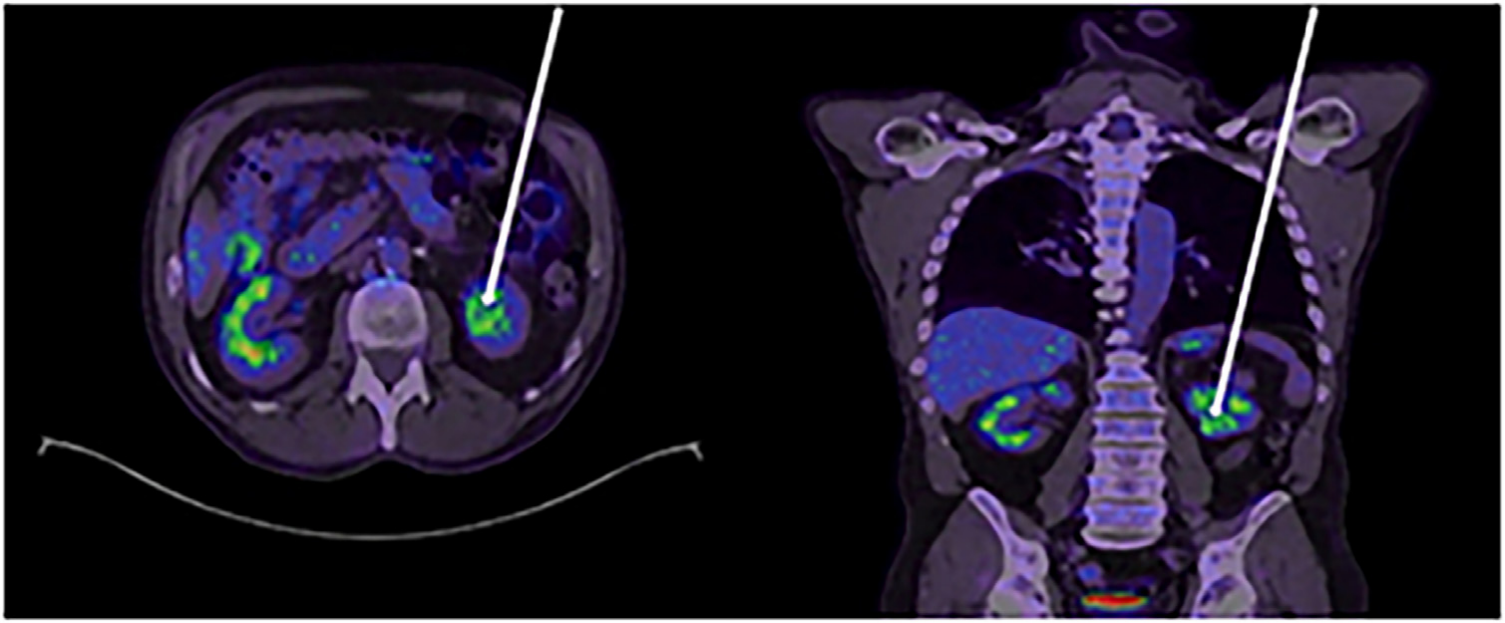
Panel 3a (axial an F-18 FDG PET CT) and Panel 3b (Coronal F-18 FDG PET CT) displaying high physiological uptake (as indicated by white arrows) in bilateral kidneys unable to clearly differentiate physiological uptake from Renal Cell Carcinoma or metastasis.

A 63-year-old male with a long term history of smoking presented with shortness of breath and new hemoptysis. CT (computed tomography) chest identified a right upper lobe mass suggestive of malignancy. Subsequent lung biopsy (Fig. 1) diagnosed squamous NSCLC. In an attempt to achieve surgical clearance, the patient then underwent upfront bi-lobectomy and right lower lobe wedge resection. Post operative histopathology confirmed NSCLC with clear margins and the patient received adjuvant cisplatin and vinorelbine. One year post surgery, stereotactic body radiation therapy (SBRT) was administered to a new right lower lobe lung lesion detected on routine surveillance imaging and concerning for oligometastatic recurrence. Following completion of SBRT a new 33mm diameter left lower pole renal mass was identified again on surveillance cross sectional imaging (Fig. 2). On non contrast imaging, the lesion was described as hypointense correlating to 30 Hounsfield Units (HU). There was minimal enhancement on contrast enhanced imaging displaying an increase of 15 HU. Additional imaging with Fluorodeoxyglucose (FDG) PET CT was unable to more clearly characterize the renal mass due to background physiologic uptake (Fig. 3).

MDT discussion recommended endoscopic biopsy, given the proximity of the mass to the renal hilum as well as the presence of significant endophytic component which were concerning for possible urothelial, renal cell carcinoma or metastatic disease.

**Fig. 4:**
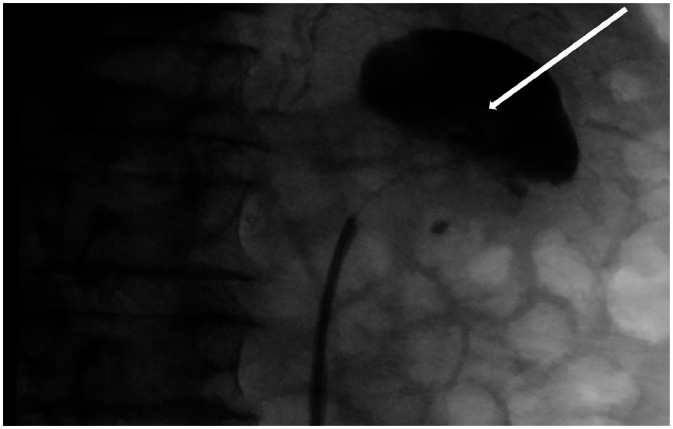
Intraoperative retrograde pyelogram displaying a dilated upper calyx with distortion of the left renal pelvis (as indicated by white arrow).

**Fig. 5:**
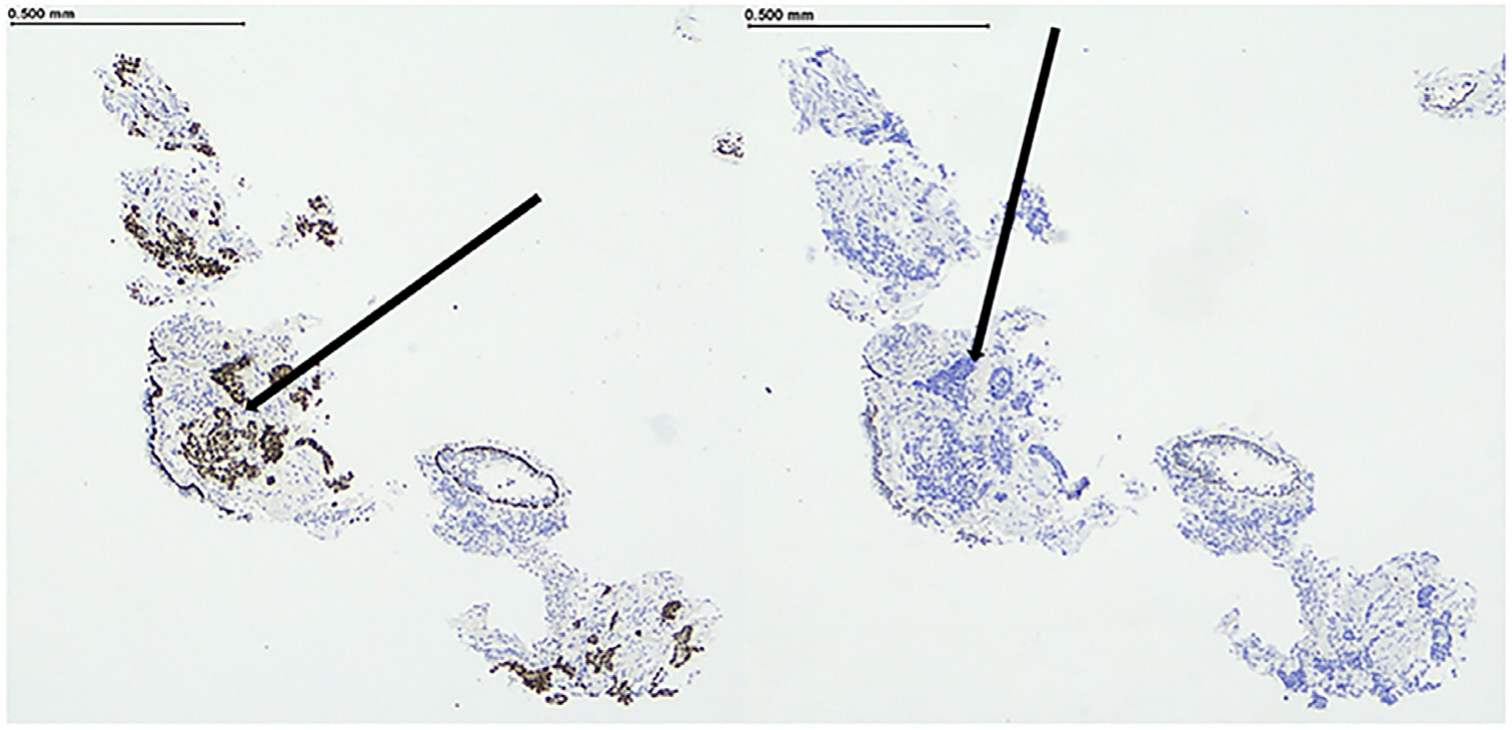
Renal biopsy: Immunohistochemistry shows the malignant cells are positive for squamous marker p40 (Panel 5a, as indicated by black arrow) and negative for adenocarcinoma marker TTF1 (Panel 5b, as indicated by black arrow).

Left retrograde pyelography revealed a distorted, compressed real pelvis with a dilated upper calyx (Fig. 4). Pyeloscopy identified papillary mucosal changes at the pelvi-ureteric junction (PUJ) so much so the flexible scope could not be passed beyond the PUJ due to macroscopic obliteration of the hilum. Non diagnostic endoscopic biopsies of the abnormal appearing PUJ and negative ureteric washings were taken at the time. A retrograde ureteric stent was also placed. Two weeks later, additional repeat endoscopic biopsies of the pelviureteric junction were once-more non-diagnostic. Percutaneous ultrasound (US) guided biopsy of the renal mass was then performed. Three 18-gauge cores were taken utilizing the coaxial biopsy technique under ultrasound guidance. Thus, an 17-gauge introducer needle was utilized necessitating only 1 puncture with 3 sequential 18-gauge biopsies then taken. No complications occurred and histopathological analysis reported metastatic recurrent NSCLC (Fig. 5).

Thus, following the incidental discovery of the renal mass on routine surveillance imaging, subsequent PET CT imaging and US guided biopsy the patient’s likely diagnosis suggested recurrent oligometastatic squamous NSCLC with renal involvement. Urological, respiratory, radiation and medical oncological opinion was again sought via the MDT. All specialist teams agreed the renal lesion was an metastatic NSCLC deposit and systemic palliative chemotherapy was recommended. Following discussion with the patient, carboplatin, paclitaxel and pembrolizumab was commenced with ongoing medical oncology follow up planned. At the time of writing this case report, the patient was receiving treatment without issue and is yet to complete any form of repeat surveillance imaging since commencing treatment.

## Discussion

Solitary renal metastasis from lung carcinoma is a rare entity with an often-poor prognosis [2]. The largest analysis to date, conducted by Adamy et al. [3] found that only 5 of 3472 patients undergoing partial or total nephrectomy across twenty years received surgery for solitary lung cancer metastasis to the kidney. There are a limited number of case reports and case series describing this clinical scenario [1,2,[4], [5], [6], [7], [8], [9], [10], [11]].

Metastatic seeding to the kidney is likely a result of the high blood flow to the kidney which accounts for 20% of cardiac output. It is thought hematogenous metastases secondary to arterial embolization is the likely mechanism of metastatic dissemination [1,12]. Given the rarity of renal metastases, particularly solitary renal metastases, an accurate and timely diagnosis can prove challenging. Patients typically present asymptomatically without hematuria or flank pain [2]. Most commonly this rare situation is identified incidentally on imaging with reportedly only 5%-10% of patients presenting with symptoms [2]. Diagnosis requires a low index of suspicion, with a particular focus on the patients clinical history in conjunction with radiological findings [4].

The diagnostic work up of most renal metastasis is initially facilitated by a multitude of imaging modalities inclusive of ultrasound, computed tomography and occasionally magnetic resonance imaging of the abdomen. Positron emission tomography (PET) may be useful for radiologically indeterminate lesions and in situations where additional metastatic disease is considered likely or will change the patient’s management [4]. However differentiating between primary renal tumors and metastatic renal masses can be challenging based upon imaging alone.

In our case report, primary TCC and RCC were known differential diagnosis. Ultrasound findings in favor of metastases typically describe homogenous, hypoechoic lesions however reports are inconsistent [13]. CT findings are also often non-specific [13]. Findings described in the literature favoring metastatic renal deposits rather than primary renal malignancy include an endophytic location, multifocality and an isodense or hypodense appearance when compared to normal renal parenchymal tissue. Metastatic renal lesions tend to enhance with intravenous contrast less than both the surrounding parenchyma and less than a primary renal cell cancer would (typically only 5-15 hounsfield units) [6]. Additional radiological descriptions on CT remain consistent, reporting metastatic renal tumors as generally multifocal, unilateral, small wedge-shaped masses which are generally located centrally within the confines of the renal capsule [4]. Typically, these lesions display an endophytic growth pattern [4]. If exophytic or cystic components are present, metastasis is unlikely and a primary RCC is more likely [14]. PET imaging itself has limited utility in the work up of most primary RCC’s and TCC’s due to high level of physiological excretion of FDG from the kidneys. This reduces the contrast between renal masses and normal parenchyma, potentially masking malignant lesions and reducing diagnostic confidence [15].

Differentiating TCC from RCC on imaging can also be difficult, particularly for centrally located renal masses such as the renal mass described in our case. Features in favor of intrarenal TCC over RCC on CT include: preserved renal shape, an focal filling defect within the pelvicalyceal system, lack of cystic or necrotic change, homogenous tumor enhancement as-well as tumor extension toward the ureteropelvic junction [16]. Apart from CT, a meta-analysis found MRI to be an useful adjunct when detecting RCC [17]. Additionally, MRI may be utilized to assist in the work up of indeterminate renal lesions or to better differentiate between the various subtypes of RCC [18]. There remains limited data on the specific MRI findings suggestive of extra renal metastasis to the kidney when compared with primary renal lesions such as TCC or RCC.

In keeping with these imaging findings, the renal mass described within our case report displayed the typical imaging characteristics (on US and CT) of a metastatic lesion within the kidney. On ultrasound the lesion was homogenous and hypoechoic and appeared endophytic. On CT the lesion was unilateral, endophytic and centrally positioned, encroaching upon the renal hilum. In addition, the pre-contrast scans showed the lesion to be hypodense (20-40 HU) with minimal to no enhancement on contrast imaging (increasing between 5 and 15 HU on the nephrogenic phase). This latter finding not in keeping with TCC. However, given the other similarities with TCC, an endoscopic ureteroscopy and biopsy was performed in our case in to assist in the exclusion of TCC as a differential diagnosis.

Making the distinction between primary and secondary tumors of the kidney is essential to guide treatment, prevent disease progression, reduce unwarranted treatment morbidity and optimize oncological outcomes. However, imaging alone is clearly unable to definitively exclude RCC or TCC, particularly in cases where endoscopic biopsy may also prove undiagnostic [19]. Thus, in addition to imaging, clinicians with a comprehensive understanding of the patient’s medical history as-well as the biological behavior of such tumors must make an accurate judgement with the clinical and radiological information available [4]. When facing diagnostic difficulty, complex decision making of this nature is best facilitated via the MDT team meeting [11]. Fortunately, the recommendation to proceed with renal biopsy following 2 unsuccessful endoscopic biopsies proved successful in our case, achieving a confirmatory histological diagnosis of metastatic squamous cell NSCLC.

Percutaneous image guided renal biopsy is a safe and commonly utilized diagnostic tool. Such biopsies are often utilized to assist in differentiating between primary and secondary malignancy [20]. The identification of patients with a metastatic renal mass remains one of the key indications for performing renal biopsy, with sensitivities of biopsy tissue reported to be up to 90% in this cohort [14]. In our case report, a single coaxial ultrasound guided renal biopsy was able to achieve what 2 attempts at endoscopic biopsies could not (a histopathological diagnosis).

However it is important to recognize that renal masses in patients with known extrarenal primary malignancies cannot be assumed to be metastases [20]. Prior studies have shown up to 57% of renal lesions in patients with concurrent extrarenal malignancy are in fact primary RCC. The majority of patients with metastatic renal lesions require systemic treatment, whereas those with a primary renal malignancy may be best treated with minimally invasive ablative treatments such as cryotherapy or surgical excision in the form of partial or radical nephrectomy [18]. Therefore, a tissue diagnosis obtained by needle biopsy provides information that can optimize the patient’s treatment [20]. However, despite the benefits, renal biopsy may return a non-diagnostic results and carry risks such as bleeding, tract seeding and vascular injury [20,21].

Amongst case reports describing solitary lung cancer renal metastases, renal biopsy appears to be utilized infrequently. Studies assessing patients with renal metastases who underwent surgery found renal biopsies prior to surgery were utilized between 3% to 45.7% of the time [[1], [2], [3],^22^]. No study has reported over 50% utilization of renal biopsy prior to surgery for a possible metastatic renal lesion. Justification for avoiding biopsy may include the potential for severe complications, with 1 study reporting massive renal hemorrhage necessitating emergent nephrectomy post biopsy [4]. There have been reported cases of multiorgan failure and progression of disease following nephrectomy where metastatic lung cancer to the kidney was only identified on post operative histopathology. Such cases argue in favor of image guided renal biopsy prior to surgery for the indeterminate lesion in order to avoid similar poor outcomes [1,4].

However, at this time there is no consensus regarding the standard of care for patients with solitary NSCLC metastases to the kidney [12]. While systemic chemotherapy remains the main treatment, surgery, radiation and cryotherapy all remain possible alternative management options [6,7,11]. The MDT plays an essential role in determining the optimal treatment strategy for complex oncological patients similar to the patient described in our case. To ensure that each patient receives the best possible management plan, we recommend all patients with potential pulmonary renal metastasis undergo an MDT conference before deciding on an approach to diagnosis and management [11].

## Conclusion

Non-small cell lung cancer rarely metastasizes to the kidney. However, despite its rarity, a high index of suspicion must be exercised for any renal mass identified in a patient with significant extrarenal oncological history. We emphasize the pertinent radiological features of metastatic renal lesion, the role of image guide renal biopsies and the MDT discussion in such settings. Finally, the pivotal role of the radiologist within the oncological MDT team meeting is illustrated.

## Compliance with ethical standards

All procedures performed in studies involving human participants were in accordance with the ethical standards of the institutional and/or national research committee and with the 1964 Helsinki declaration and its later amendments or comparable ethical standards.

## Author contribution

All authors contributed toward the writing, analysis, drafting and revision of this paper and gave final approval of this version to be published. All authors have agreed to be accountable for all aspects of this work.

## Patient consent

All authors confirm that a written, informed consent for publication of this case was obtained from the patient.
